# Ethics and health research priority setting: a narrative review

**DOI:** 10.12688/wellcomeopenres.21182.1

**Published:** 2024-04-17

**Authors:** Joseph Millum

**Affiliations:** 1 Philosophy, University of St Andrews, St Andrews, Scotland, KY16 9AL, UK

**Keywords:** Ethics, equity, research priority setting, research agenda setting, health policy

## Abstract

This narrative review aims to describe current practice and ongoing discussions in the academic literature regarding ethics and health research priority setting. It begins with some preliminary distinctions regarding types of research priority setting. It then gives some background on current practice with respect to formal research priority setting exercises, including summaries of The Ad Hoc Committee on Health Research method, the Child Health and Nutrition Research Initiative (CHNRI) method, the Combined Approach Matrix (CAM), the Delphi method, the Essential National Health Research (ENHR) strategy for priority setting, and the James Lind Alliance (JLA) framework. The majority of the paper reports the results of a literature review covering specifically ethical issues under the thematic headings of *process criteria*, *substantive criteria*, *global justice*, the *obligations of specific actors*, and *research topics*. It closes with some summary thoughts about apparent gaps and directions for future investigation.

## Plain language summary

More health research is needed to develop new treatment and prevention options for many diseases. But there are limited resources available to support health research. This means that difficult decisions must be made about how to allocate those resources among competing important projects. Making these decisions is called *priority setting*. Dr. Joseph Millum reviewed what has been published on the *ethics* of health research priority setting. First, he compared different methods that have already been developed to help organizations and governments set priorities. Second, he identified themes in the current discussions about ethics and priority setting. Some important themes included: how stakeholders should be included in priority setting exercises; what would be a fair allocation of research resources; global disparities in health research; and how different types of funder should think about their obligations. The results of this review will inform guidance from the World Health Organization on how to incorporate ethics into health research priority setting.

## 1. Introduction

There are always more valuable potential health research projects that could be carried out than there are resources to support them. The organizations and individuals that make decisions about what health research (henceforth “research”) gets done therefore have to make difficult decisions. In addition to the technical challenges involved in working out which projects are likely to succeed, at what cost, and with what results, research priority setting has an ethical dimension. Though the results of research necessarily cannot be predicted with certainty, which research gets done does predictably affect who will benefit from new knowledge. Studying the genetics of cancer is more likely to lead to benefits for cancer patients than for pneumonia patients; studying health care delivery systems in Bangladesh is more likely to ultimately benefit Bangladeshis than to benefit Italians. Decision-makers allocating scarce research resources can—and should—ask how these resources should be *fairly* allocated among potential beneficiaries.

Modern discussion of the ethics of research priority setting can be traced back to the Commission on Health Research for Development’s 1990 report, *Health Research: Essential Link to Equity in Development*. This report identified, “a gross mismatch between the burden of illness, which is overwhelmingly in the Third World, and investment in health research, which is overwhelmingly focused on the health problems of the industrialized countries.” ^1^ The mismatch was labeled the “10/90 gap.” ^2^ This blatantly unjust allocation of global health research resources provoked multiple critiques of how decisions about research are made, as well as concrete proposals for how they could be made in a more equitable way. ^3^ But although the global allocation of research resources is one natural target for criticism, there is no single body responsible for setting global research priorities. There are many actors involved in research priority setting whose decisions are amenable to ethical evaluation. A wide range of funders—from government bodies and multilaterals, to non-profits and foundations, to for-profit companies—make decisions about what research to support. Researchers and universities also make decisions about what projects to pursue. Within the constraints of what they can get funding for, they have considerable latitude to investigate what they regard as important. Community and patient advocacy organizations attempt to influence the research agenda to support specific populations and conditions. Academic journals, too, affect what research is conducted via the signals they give about what research they will publish. Finally, there are many governmental and international bodies that affect research priorities, either directly or indirectly. For example, governmental bodies directly fund research. Indirectly, they also affect what research is conducted through health care systems that pay for certain products of research, by training future researchers, by investing in infrastructure, and by setting research agendas that others are encouraged to follow.

This narrative review aims to describe current practice and ongoing discussions in the academic literature regarding ethics and health research priority setting. It begins by giving some background on current practice with respect to formal research priority setting exercises. It then reports the results of a literature review covering specifically ethical issues under thematic headings. The paper closes with some summary thoughts about apparent gaps and directions for future investigation. This review is part of a larger World Health Organization project on the ethics of health research priority setting. That project aims to describe the ethical considerations relating to the allocation of scarce resources for health research and guide key decision-makers in incorporating these ethical considerations into their work. Among other tools, it will develop ethics guidance for the various actors who allocate scarce resources for health research.

## 2. Current practice

### 2.1. Preliminary distinctions

Any organization or individual who makes decisions that aim to affect what health research is conducted is involved in setting health research priorities, since they are thereby allocating—or recommending the allocation of—a scarce resource. Research priority setting takes quite different forms, which can be helpfully described along several dimensions. ^4^

First, priority setting can involve allocating—or recommending the allocation of—very different *types of resource*. It is natural to think in terms of funding, but what research is conducted is also affected by the time allotted, available research infrastructure (e.g., MRI machines), eligible research participants, and even trainee places.

Second, we can distinguish *explicit* and *implicit* priority setting. Explicit priority setting is conceptualized as such (so that the decision-makers conceive of what they are doing as allocating a scarce resource, setting priorities, or developing a research agenda). But other decisions that affect the allocation of health research resources are not explicitly described as priority setting. For example, a funder may say that they are just funding the best science or a researcher may say that they are just investigating an interesting question. Still, they have made decisions about how resources will be allocated that affect who benefits from the research.

Third, priority setting may be more or less *direct*. A funder that decides which applications get funded is allocating resources directly. Likewise, a researcher making decisions about how to spend research time that is supported by a university salary (i.e., their “hard money”). On the other hand, an advocacy group campaigning for more funding to be directed to patient research priorities is engaged in indirect priority setting. Likewise, a journal that encourages submissions on particular “high-priority” topics.

Fourth, the *scope* of priority setting may differ. Formal priority setting exercises often take a geographical or topical scope. For example, a national body might seek to establish priority research areas for the country, or an academic society might publish results of a global priority setting exercise for their discipline. The scope might be even more limited—for example, to research carried out at a particular institution, ^5^ or to decisions about which trials to support and which to close. ^6^ Or, the scope could be much broader, as with the analysis supporting the criticism of the “10/90 gap.”

A number of methods have been developed to assist with explicit research priority setting exercises. In what follows, I provide a brief overview of the methods most often used or referenced in the literature. I then summarize reviews of the use of such methods.

### 2.2. Formal methods for priority setting exercises

The methods described here are the six that were most frequently mentioned in reviews of health research priority setting in practice ( Section 2.3) and in the literature on ethics and research priority setting ( Section 3). Methods not summarized here for reasons of space include Listening for Direction, ^7^ the adaptation of the Choosing All Together (CHAT) exercise to health research, ^8^ and value of information analysis. ^9^ There are also several manuals that provide guidance in setting up and managing a priority setting exercise that include overviews of common priority setting methods and their pros and cons. ^10^ In addition, Elizabeth Manafò *et al.* provide a helpful review of patient and public engagement methods for priority setting exercises from a set of high-income countries. ^11^

Several questions must be answered in order to carry out a priority setting exercise. These include:

1. How will candidate priorities (i.e., the research options to be prioritized) be identified?
2. What criteria will be used to rank the candidate priorities?
3. Who will be involved in the exercise and what is their role?

The methods summarized here provide different answers to these questions. However, most are deliberately incomplete. For example, all defer to some extent to the organizers of and/or participants in a priority setting exercise with respect to the criteria used for ranking candidate priorities. Likewise, they give varying degrees of leeway to the organizers regarding who is involved and what role the participants play. Further, as will become apparent, though each method is designed to help set priorities in a systematic way, the methods vary substantially in their approach. Some primarily deal with how to gather and synthesize information about research options. Others are more concerned with the process for bringing stakeholders together to rank priorities. Finally, the documents that describe these methods all make some suggestions about values—for example, through assertions about the goal of priority setting, or regarding what constitutes a fair process. I summarize what they say about values in Section 3 with the rest of the review of the ethics literature on research priority setting.

***The Ad Hoc Committee on health research method.*** I begin with the method recommended by the WHO’s Ad Hoc Committee on Health Research Relating to Future Intervention Options in 1996. This method, though incomplete, is cited by and was built upon by later designers of research priority setting methods. It involves technical experts answering five questions with respect to a health problem in order to assess research gaps and the relative priority of the problem:

1. How big is the health problem?
2. Why does the disease burden persist?
3. Is enough known about the problem now to consider possible interventions?
4. How cost-effective will these interventions be? How quick and how expensive to develop?
5. How much is already being done? ^12^

**Figure 1.**
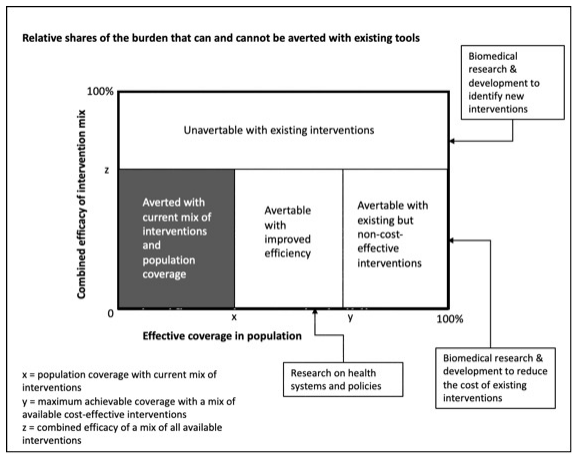
Analyzing the burden of a health problem to identify research needs. (Adapted from WHO 1996: 7).

Figure 1 reproduces the committee’s helpful representation of how understanding the causes of disease burden and the cost-effectiveness of existing interventions can guide research priority setting.

With respect to the three questions identified above:

1. Candidate priorities are identified through answering the committee’s five questions.
2. The main criterion for ranking candidate priorities is the expected cost-effectiveness of resulting reductions in global disease burden.
3. Implicitly, priority setting is to be carried out by individuals with technical expertise in the relevant subject matter. This question is not directly answered.

***The Child Health and Nutrition Research Initiative (CHNRI) method*.** As its name suggests, the CHNRI method was originally developed by researchers looking to set priorities for research into child health and nutrition. However, there is nothing in the method to prevent it from being applied outside of child health, and, indeed, it has been used in a variety of contexts, including for sexual health, mental health, and infectious diseases. ^13^ The CHNRI process involves a technical working group who define the context for the priority setting exercise (e.g., target population, target disease burden). They survey a large number of experts to identify the candidate priorities. These experts are typically researchers with subject matter expertise, but could include policy makers and program managers, depending on the context. This process yields “an exhaustive list of the competing research options by addressing main risk factors and possible interventions.” ^14^ Once the list has been consolidated by removing duplicate ideas and integrating related ideas, the experts are asked to score each candidate priority. CHNRI suggest five “standard” criteria for prioritization among research options: (i) answerability, (ii) effectiveness, (iii) deliverability, (iv) maximum potential for disease burden reduction, and (v) effect on equity. However, the specific criteria used can be changed, if the organizers so decide. Finally, the criteria can be weighted on the basis of surveying external stakeholders from the wider community. By assigning the criteria different weights, non-experts thereby provide input with regard to what they value more or less. Those weights are applied to the experts’ scores to give a ranking of all the candidate research priorities.

With respect to the three questions identified above:

1. Candidate priorities are identified by surveying experts (usually researchers).
2. Five standard criteria are used to rank the priorities, though the organizers have the option of using different criteria.
3. Funders or government bodies may be involved in setting up the priority setting exercise and deciding on the context (i.e., the parameters of the exercise) and possibly the criteria to be used. Subject matter experts identify the candidate priorities and rank them. Other stakeholders are involved only through suggesting weights for the criteria.

***The Combined Approach Matrix (CAM)*.** The Combined Approach Matrix (CAM) is a tool with which to “classify, organize and present the large body of information that enters into the priority setting process.” ^15^ This method starts with an individual or group of experts completing a matrix, which can have either two dimensions ( *public health* and *institutional*) or three (adding an *equity* dimension). The public health dimension captures information on disease burden, determinants of disease, the present level of knowledge about disease, the cost and effectiveness of existing interventions, and current resources flows. The institutional dimension then allows the public health information to be assigned to different levels: individual, household, and community; health ministry and other health institutions; non-health sector; and governance. The equity dimension—a more recent addition to CAM—involves assessing whether there are differences between social groups, such as gender and income groups. Completing this matrix allows knowledge gaps to be identified, which might themselves be research priorities, as well as presenting all the relevant information relating to a priority setting process in a systematic way.

It should be noted that the CAM is not itself sufficient to constitute a priority setting method. Its authors recommend using the CAM as part of “an interactive workshop that involves all the relevant stakeholders in decision-making.” ^16^ Exactly how that workshop should be planned and run is left up to the organizers.

With respect to the three questions identified above:

1. Candidate priorities are typically identified by experts (e.g., through consulting subject matter experts or reviews of the literature).
2. The criteria for ranking are not specified, though implicitly they include potential reduction in disease burden, cost, and equity considerations.
3. CAM does not specify how decisions get made, so only the involvement of experts in the creation of the matrix is dictated by the method.

***The Delphi method*.** The Delphi method is a systematic way to gather information and opinions from a group of experts. Originally developed for forecasting, it is used widely to guide decision-making in situations where there is insufficient knowledge for evidence-based modelling, including for research priority setting. ^17^ There are several variations on the method, but the basic idea involves convening a panel of experts who are asked to answer questions or rank options. Their responses, including their reasoning, are compiled and then circulated to the same panel, which repeats the exercise one or more times. ^18^ A Delphi exercise may aim for consensus or simply aggregate the panel members’ scores. 

With respect to the three questions identified above:

1. Candidate priorities are typically identified by the experts (but could be provided to them by the organizers of the priority setting exercise).
2. Use of the Delphi method does not entail the use of any specific criteria.
3. Subject matter experts identify the candidate priorities and rank them.

***The Essential National Health Research (ENHR) strategy for priority setting*.** The ENHR strategy was designed by the Council on Health Research for Development (COHRED) for country-level priority setting exercises. ^19^ The method centers around a national workshop. The team convening the priority setting exercise first sets up a working group of stakeholders who decide how the priority setting exercise will be run and carry out a “situation analysis” to provide the necessary background data. ^20^ An initial list of research ideas is generated through the “situation analysis and inputs from various stakeholders.” ^21^ The criteria and scoring method used to rank these ideas are agreed upon by consensus.

The workshop itself involves a larger group of stakeholders, who are supposed to represent the various groups with interests in health research in the country. The participants in a workshop:

represent ENHR stakeholders, i.e., researchers, decision-makers at various levels, health service providers and communities. Private sector participants are equally important (for example, professional associations or the pharmaceutical industry), as well as parliamentarians, potential donors and international agencies. ^22^

This larger group scores the research ideas using the criteria and scoring system agreed upon. The result is a national research agenda. 

With respect to the three questions identified above:

1. Candidate priorities are identified through the working group’s situation analysis and inputs from stakeholders.
2. The criteria used to rank candidate priorities are decided on by the working group. The ENHR manual suggests 28 possible criteria falling into four categories: *appropriateness*, *relevancy*, *chance of success*, and *impact*.
3. A small working group of stakeholders is involved in designing the process to be used and a larger group in scoring the candidate priorities. The guidelines emphasize being as inclusive as possible.

***The James Lind Alliance (JLA) framework*.** The James Lind Alliance, “brings patients, carers and clinicians together in Priority Setting Partnerships (PSPs) to identify and prioritise the unanswered questions or evidence uncertainties that they agree are most important for research to address.” ^23^ They deliberately exclude “representatives of the pharmaceutical industry, other commercial businesses, or those in the research community who are not also clinicians, patients or carers.” ^24^

The JLA method begins with the organizers of the PSP gathering “uncertainties” from existing guidelines and systematic reviews, and surveys of patients or service users, carers, and clinicians. The uncertainties are checked for whether they are in scope, to remove overlap, and stated in the form of “indicative questions.” Once questions that already have answers are removed, this typically results in a long-list of 60–70 questions.

An interim priority setting process then takes place through a survey of stakeholders to whittle the long-list down to 20–30 questions. Survey responses from patients and carers are equally weighted to responses from clinicians. Finally, the process culminates in a workshop with 12–30 participants—again drawn from patients, carers, and clinicians. Through small group discussion and ranking exercises, the participants develop consensus on a top-10 list, where: “The aim of the Top 10 is to highlight important areas for research, but not necessarily to come up with the specific research questions.” ^25^

With respect to the three questions identified above:

1. Candidate priorities are identified through examining existing guidelines and systematic reviews, and surveys of patients or service users, carers, and clinicians.
2. The candidate priorities are ranked by patients, clinicians, and carers.
3. The PSP is led by a steering group which includes patients, carers, clinicians, and a JLA Adviser. Surveys, interviews, and workshops participation is restricted to patients, clinicians, and carers.

### 2.3. The use of priority setting methods

Reports of priority setting exercises are mostly either regional (typically national but sometimes a larger region) or by health topic (e.g., diabetes, emergency medicine). Reviews of these priority setting exercises suggest that current practice varies widely in its scope and in the use of priority setting methods. 

Robert Terry *et al.* conducted a review of “research priority setting undertaken by the technical programmes based at the WHO headquarters in Geneva from 2002 to 2017.” ^26^ From 116 documents, 2145 research priorities were extracted, addressing 73 diseases or health topics. Most used expert consultation, often, but not always, in conjunction with a literature review. The authors report:

“Of the 2145 priorities in this dataset, few were generated using a published research prioritisation framework, such as Child Health and Nutrition Research Initiative (n = 30), Multiple Criteria Decision Analysis (n = 132) or Delphi (n = 107). None of the priorities were generated using the COHRED 3D method [CAM].”

Outside of WHO, Sachiyo Yoshida conducted a literature review looking at the methods used in published health research priority setting exercises between 2001 and 2014. Of 165 studies, 26% used the CHRNI method, 24% the Delphi Method, 8% the James Lind Alliance method, 2% CAM and one study used ENHR. ^27^ She concluded that the number of exercises is increasing with time, as is the use of formal priority setting methods.

Reviews of national health priority setting describe wide variation in methodologies and documentation. For example, Tomlinson *et al.* reviewed nine country-level priority setting exercises in LMICs. ^28^ Most used a systematic priority setting method, such as the CHNRI method or CAM. Stakeholder involvement was variable and sometimes very limited. Documentation of the process also varied substantially. Ludovic Reveiz *et al.* conducted a systematic review of priority setting methods used in Latin America and the Caribbean. ^29^ Thirteen of 18 Latin American countries, plus the Caribbean Health Research Council had documented health research priorities. Relatively few countries used formal priority setting methods and there was little planning for implementation or evaluation of the effects of the priority setting process. Nonetheless, a variety of stakeholders were included, some countries engaged in public consultation, and the results of the priority setting exercises were publicly available.

Finally, Kristina Staley and Bec Hanley for the James Lind Alliance conducted a scoping review of whether and how UK-based research funding bodies set priorities. Among those who reported setting priorities, the methods used vary widely—from surveys and focus groups to adopting expert recommendations. Dedicated funding was sometimes set aside for priorities, but for the most part these organizations funded research proposals according to peer review assessments of scientific quality. Notably, “None of the 22 organisations we interviewed make an assessment of ‘how well a proposal fits with a research priority’ a major or explicit part of the decision about which projects to fund.” ^30^

## 3. Ethics and health research priority setting

### 3.1. Methods

For this topic, I aimed to conduct a more comprehensive review of the published English-language literature on ethics and health research priority setting. I began with a search of PubMed using a search string adapted from Christiane Grill’s recent scoping review of involvement of stakeholders in research priority setting: ^31^

(“research priorit*” OR “priority research” OR “research agenda setting” OR (“agenda setting” AND “research”) OR (“agenda setting” AND “priorit*”) OR (“research agenda AND priorit*”)) AND (equit* OR ethic* OR bioethics)

This yielded 1201 results. From these I removed those clearly out of scope given title and abstract snippet. That left 95 documents whose full abstracts I reviewed and screened out 51, leaving 44 documents for the literature review. As well as literature that was clearly irrelevant, I excluded reports of priority setting exercises, discussions of health *care* priority setting, and documents on topics in research ethics (including discussions of whether a particular type of research is ethical at all, e.g., cloning research, and discussions of the inclusion or exclusion of particular populations as research participants, e.g., involving pregnant women as research participants). Commentaries on other articles were also excluded. As well as documents explicitly concerned with *ethics* and research priority setting, I included those on the topic of research priority setting that addressed community engagement, underserved communities, stakeholder values, and similar ethically salient topics.

Since PubMed does not include all bioethics or philosophy journals, this was supplemented by a research assistant (Anna Videbaek Smith) with a search of Google Scholar and PhilPapers for other relevant works. The Google Scholar search combined variations of “health research priority setting,” “health research allocation,” “health research agenda,” “health research funding,” and “health research prioritisation” with “ethics,” “ethical,” and “moral.” The PhilPapers search did the same but omitted the qualifiers of “ethics,” “ethical,” and “moral.” A review of paper titles and abstracts yielded 42 documents. I reviewed these to remove those out of scope or meeting exclusion criteria. The remaining 20 were included in the final set.

Finally, the results of these searches were augmented with publications from the author’s own collection from earlier research. This added a further 25 publications that had not been already identified through the database search.

I read all 89 documents, summarized them, and extracted themes.

### 3.2. Results

I classified the themes that I identified into the categories of *process criteria*, *substantive criteria*, *global justice*, the *obligations of specific actors*, and *research topics*.

***3.2.1. Process criteria*.** A great deal of the literature focuses on the processes by which research priorities are set. There is general agreement on the importance of involving “stakeholders” in these processes. There appear to be differing views about who should be involved and how.

Stakeholder groups that are mentioned include: patients, patients’ families, carers, communities, health service providers, researchers, policy makers, elected representatives, professional associations, NGOs, pharmaceutical companies, donors, and international agencies. ^32^ Some guidance documents recommend maximal inclusiveness; ^33^ others are more selective because they want to get the perspectives of specific groups, such as patients and clinicians, who might otherwise not be heard. ^34^

There is a lot of discussion of how stakeholders should be involved and, in particular, the ways in which a priority setting process may fail to include stakeholders from more disadvantaged groups in meaningful ways. Bridget Pratt writes:

Engaging communities that are considered disadvantaged and marginalized in priority setting is essential to making their voices and concerns visible in global health research projects’ topics and questions. However, without attention to power dynamics, their engagement can often lead to presence without voice and voice without influence. ^35^

According to Pratt *et al.*, equity-oriented research priority setting required “deep inclusion.” ^36^ Their model of inclusion has three dimensions: breadth, qualitative equality, and high-quality non-elite participation. *Breadth* refers to the *range* and *mass* of participants who are included in priority setting—that is, the different groups who are represented and the number of individuals from each group. *Qualitative equality* means equality in the ability of participants to influence the priority setting process, so that more powerful individuals do not have greater influence. The *quality* of *non-elite participation* ranges from consultations to elicit feedback or information through to full partnership involving shared decision-making throughout the priority setting process.

Several publications describe deficiencies in the way particular groups are included in priority setting exercises. It is reported that researchers and research funders are consulted but that the views of community members and patients are either not elicited or are not heeded to the same extent. ^37^ Even when community organizations partner with researchers, ideal levels of breadth, qualitative equality, and non-elite participation may not be attained. ^38^

One response to the perceived lack of voice and influence by certain stakeholder groups has been to design priority setting processes around their inclusion. ^39^ For example, some priority setting exercises have been carried out with only members of less-advantaged communities, rather than including other stakeholder groups. ^40^ Others focus on the patient experience and aim to elicit what patients think is important in terms of research to improve their health or their health care experience. ^41^ Consideration of patient preferences was written into the legislation establishing the Patient-Centered Outcomes Research Institute in the US. ^42^ What members of “minority and underserved communities” think should be health research priorities and the criteria that should be used to compare research options were explored in recent qualitative studies. ^43^ Finally, as described above, the JLA framework for priority setting is also designed to elevate patients’ voices and prioritize the patient experience. Their PSPs include only individuals with direct experience of target health conditions: patients, family members, carers, and clinicians.

The questions of *who* should be included in research priority setting and *how* they should be included are likely best answered by addressing the prior question of *why* a group should be included. A range of reasons for including stakeholder groups are suggested in the literature (though the connections to who should be included and how are not always clearly drawn). They can be divided into *instrumental* reasons and *intrinsic* reasons for inclusion. There are *instrumental* reasons to include members of a group if doing so will produce better results and there are *intrinsic* reasons if it is valuable to include them independent of the effect of doing so on the outcome of priority setting.

Among instrumental reasons we can further distinguish epistemic and non-epistemic reasons for inclusion. Start with epistemic reasons—that is, reasons for inclusion that have to do with gaining knowledge. The best way to find out what matters to patients, for example, is usually to ask them. This is a reason to include patients in a priority setting process and to do so in a way that elicits information about their experiences of their disease, their treatment, and so on. ^44^ It is also a reason to include, as far as possible, patients who represent different manifestations and stages of a disease, different demographic groups, and so on. ^45^ Likewise, there are epistemic reasons to include subject-matter experts—like scientists—in priority setting. They are likely to be best placed to identify unanswered research questions and to assess how feasible answering those questions would be. ^46^ A number of authors also propose that stakeholders decide on the criteria that should be used for ranking different research options. ^47^ These stakeholders could be surveyed independently or could simply be the participants in a priority setting workshop. Little argument is generally given for why such *value* judgments should be made in this way, though some suggest that it is an appropriate response to uncertainty or disagreement about the correct criteria to use. ^48^

Non-epistemic instrumental reasons for stakeholder involvement in priority setting include building trust with patients and communities, ^49^ getting uptake of the reported priorities from decision-makers and end users of research, ^50^ promoting social justice, ^51^ and building research and priority setting capacity. ^52^

Some authors also give intrinsic reasons for including certain groups in priority setting. Where a priority setting process is led by a government (or makes recommendations to government) it is sometimes suggested that citizens have a right to be represented. ^53^ Even outside of government activities, some suggest that those who are affected by research decisions ought to be included—perhaps out of respect or for reasons of epistemic justice. ^54^ For international research, the legacy of colonialism casts a long shadow. The critiques developed by authors who address the decolonization of health research might provide further intrinsic reasons to take into account the viewpoints of people from formerly colonized nations, as well as to share decision-making power with them. ^55^

Outside of formal priority setting exercises, concerns about the legacy of colonialism and imbalances of power also arise within research collaborations. LMIC researchers who work with HIC researchers and institutions report a variety of issues. They may be excluded from decisions about what research is done or their inclusion may be tokenistic, so that they lack genuine power. Instead, priorities are set by HIC researchers or by international funders. ^56^ Among other things, this can lead to misalignment between the goals of the research projects and local or national priorities. Within ongoing research projects, LMIC researchers may find themselves, “relegated to the role of ‘a glorified field worker’ … That is, of being seen as responsible for providing samples but being excluded from the creative, interesting and ‘scientific’ features of the collaboration.” ^57^ Inequitable partnerships are not inevitable, however. For example, Maarten Olivier Kok *et al.* describe the Ghanaian-Dutch Health Research for Development Programme, which funded 79 studies between 2001 and 2008. It was designed as a “programme for demand-driven and locally led research in Ghana” with Ghanaian lead investigators responding to Ghana’s national research priorities. ^58^

In addition to criteria relating to inclusion and power-sharing, other ethical criteria related to fair processes for research priority setting are mentioned in the literature. A number of authors mention the importance of a *transparent* process, which helps to ensure integrity, accountability, and potentially the replicability of a priority setting exercise. ^59^ Some cite the “accountability for reasonableness” (A4R) framework that was developed by Norman Daniels and James Sabin for health *care* priority setting. ^60^ According to A4R, a fair process for priority setting should meet requirements of publicity, relevance, and revisability. ^61^ Finally, several writers call attention to the fact that decisions about the allocation of research resources are often political, at least in part. ^62^ This is a fact that needs to be acknowledged pragmatically, even if it would be better were it not the case.

***3.2.2. Substantive criteria*.** The outcomes of priority setting—that is, possible allocations of resources among research projects or rankings of research options—are open for ethical evaluation, as well as the process. I label the criteria that are used to evaluate outcomes *substantive criteria*, in contrast to *process criteria*. Though much of the literature on the ethics of health research priority setting is focused on process criteria, there is still considerable discussion of substantive criteria.

It is striking that there is near-universal agreement on two ultimate goals for research priority setting: the improvement of population health or wellbeing and the fair distribution of benefits. ^63^ These twin goals are stated in different ways by different authors, which suggest different ways in which they might be conceptualized. For example, Nicola Barsdorf and Joseph Millum write:

… the social value of research should be conceptualized as a function of two considerations: 1) the expected benefits of the research project; and 2) the degree of disadvantage of the expected beneficiaries of the research project. The more benefit that is anticipated from a research project, the higher its social value; the more disadvantaged the beneficiaries of a research project, the higher its social value. ^64^

Yvo Nuyens makes the point as follows:

… the goal of any activity to set priorities for health research is to define an investment portfolio of health research and development that will have the greatest possible impact on the health of the majority of the population, in particular those who are poorer. ^65^

Differing conceptions of these shared goals leave open the answers to several important questions. One concerns the metric of advantage. Should those allocating scarce resources for health research aim to promote health, wellbeing, or some other measure of value? This topic has been discussed in the context of priority setting for health *care*, ^66^ but did not receive much attention in the health research priority setting literature reviewed here. Another question concerns how the two goals should be balanced against each other. For example, how should we trade off a gain in population health against a reduction in health inequality? This issue is also not directly addressed in the literature.

Some documents do touch on the question of how the fair distribution of benefits should be conceived. They often use the terminology of *equity*. Where health equity is given a clear meaning, it is usually defined in terms of the absence of avoidable differences in health status between social groups. ^67^ Relatedly, some writers speak in terms of giving higher priority to those who are worse off or more disadvantaged. ^68^ Others connect health research to the requirements of social justice. ^69^ For example, Danielle Wenner argues that:

Clinical research is one aspect of an institutional structure that governs the health systems that are available to individuals, that individuals cannot opt out of, and that will have deep and lasting impacts on their life prospects, their final ends and purposes, and the way that they think of themselves. ^70^

Wenner concludes that the connection between clinical research and the “basic structure” of society means that “all individuals have standing to claim a voice in biomedical research priorities,” as a matter of justice, where this includes the priorities of private for-profit enterprises, not just government-supported research. ^71^

Several documents specifically mention *cost* as a criterion that should be taken into account in priority setting. ^72^ This is consistent with the idea that allocators should seek the greatest impact in terms of population benefits and equity, given the limited resources available to them. ^73^ Proponents of the use of *value of information analyses* for research priority setting propose quantifying the cost-effectiveness of research by tying the information gained from the research directly to the decisions that information will improve within health care systems. ^74^ In principle, at least for certain types of research within a single health care system, decisions about how to spend money on research could then be integrated with decisions about spending on care.

Further questions of justice or fairness arise in two other discussions in the literature. First, they arise in discussions of “orphan diseases.” ^75^ These are conditions that will be under-researched by for-profit entities under standard market conditions. Some will be under-researched because they are *rare*. If there are too few potential beneficiaries, then the research and development costs of a new drug may be unlikely to be recouped. Other conditions will be under-researched because they are mostly found in patient populations who are unable to afford patented interventions, such as snakebite, leprosy, and dengue fever. These conditions are neglected simply in virtue of being rare in wealthy populations. The ethical argument in favor of providing more support for common but neglected diseases is straightforward: it is unfair for a population to have worse access to health care because it is poorer. The practical question of how to motivate research for these diseases remains. ^76^ The ethical argument in favor of providing more support for research into rare diseases is less straightforward. Some argue that it is unfair to patients with rare disease if those diseases do not get researched. ^77^ Others suggest that there may be greater scientific opportunities from researching rare diseases, even if the patient population that stands to immediately benefit is small. ^78^ In a number of jurisdictions, including the European Union, Japan, and the United States, legislation has been enacted to incentivize for-profit research into rare diseases. ^79^

Second, there are discussions of whether research resources are distributed in a way that is appropriately proportional to need. For example, public funders of research have come under considerable criticism for the apparent mismatch between levels of funding and the burden of disease in a population. ^80^ These critics appear to think that it is unfair when funding is not *proportional* to disease burden. ^81^ Joseph Millum analyzes this critique and concludes that a version of it is justified. He argues that, “diseases that are globally under-funded are those that receive a smaller fraction of total funding, conditional on scientific opportunity, than their severity-weighted contribution to the global burden of disease.” ^82^

A different take on proportionality looks at the distribution of funds across types of research, rather than across diseases. For example, Tikki Pang highlights concerns that research funding—including from non-profit and governmental funders—is skewed towards developing new technologies, rather than focusing on the delivery and use of existing technologies. ^83^ Similarly, Timothy Krahn and Andrew Fenton criticize the Canadian government’s funding of autism spectrum disorders (ASD) research, which they regard as too heavily focused on biomedical and clinical research to the detriment of health systems and population health research. ^84^

A distinct type of substantive criterion is the *constraint* or *screening criterion*. Some research should not be conducted—for example, because it cannot be carried out without unacceptable risks to research participants or third parties. Some of the documents describing methods for research priority setting emphasize the importance of identifying such criteria, in order to rule out certain research options from the beginning. ^85^

Finally, many writers issue cautionary notes about the ability of those setting priorities to accurately assess the effects of decisions about what research gets done. Much depends on scientific serendipity and so cannot be predicted in advance. Barry Bloom *et al.* write:

A key challenge is the problematic nature of anticipating scientific connections in advance. For example, the sequencing of a mouse leukemia virus genome as part of the National Cancer Program is what enabled scientists years later to classify HIV as a related member of the retrovirus family. Indeed, who would have predicted that research on the once arcane coronavirus would become essential to control the spread of SARS? Or that the esoteric question of whether tumor cells extinguished differentiated functions of normal body cells would lead to the discovery of monoclonal antibodies? Or that the study of sex in bacteria would give rise to the entire genetic revolution of the past half century? ^86^

***3.2.3. Global justice*.** Ethical issues concerning global justice are frequently mentioned in the literature. Many are related to the apparent maldistribution of resources whereby the majority of health research funding is spent developing medical technologies for conditions experienced by wealthy populations (the “10-90 gap” described above). ^87^ This maldistribution is criticized not merely because of the neglect of health conditions primarily experienced by poorer populations, but because of the emphasis on drugs and other marketable technologies. Some writers argue that there should be a greater focus on research on health systems and the social determinants of health. ^88^ Such research would be more likely to meet the needs of populations in LMICs, even if it would not be as profitable.

The majority of health research funding globally comes from HIC institutions, including pharmaceutical companies, governmental funders, and non-profits. The resulting imbalances in power and resources can also be viewed through a historical lens. It reflects, still, the legacy of colonialism—those countries with the greatest influence over what research is conducted globally are frequently those who were engaged in colonial or neo-colonial domination, extraction, and oppression. As described above, this requires researchers and decision-makers from HICs to be particularly attentive to power relationships in research partnerships, to ensure that the voices of LMIC populations are heard and have influence in research priority setting, and be alert to possible epistemic injustices. ^89^

A further question concerns the relationship between priorities that are set at different levels. For example, international funders or global bodies (such as the WHO) may establish what they consider global priorities, say for diabetes or nutrition research. But national bodies may also set priorities for research within a specific country. These priorities may then clash. In such cases, should national priorities drive global priorities or vice versa? Though some support the idea that national priorities should direct global priorities, ^90^ the issue is complex and the appropriate relationship between different levels of priority setting may depend on the context. ^91^ At a minimum, it is agreed that there should be support for research that is responsive to local needs in LMICs. Several authors argue that international funders and research groups should build local research capacity, which would then enable LMIC researchers to pursue research that they judge to be of local importance. ^92^

One important driver of the “10–90 gap” is the international patent system. A patent is a form of intellectual property that grants the patent-holder a temporary monopoly over the right to manufacture the patented entity. Patents are granted over inventions that meet specific criteria, including novelty, utility, and non-obviousness, provided that the subject matter is patentable. The temporary monopolies provided by patents on drugs and medical devices allow the patent-holders to charge prices far above production costs and thereby reap substantial profits. Their proponents justify patent protections on the grounds that the prospect of these profits incentivizes private actors—such as pharmaceutical companies—to expend the considerable sums necessary to develop new interventions. Absent such incentives, it is argued, there would be little motivation to conduct such research, since other manufacturers would be able to free ride on the research by copying successful products. ^93^ In 1995, the World Trade Organization’s Agreement on Trade-Related Aspects of Intellectual Property Rights (the “TRIPS agreement”) came into force. It standardized intellectual property protections internationally, including requiring that national legislation provide for at least twenty-year product patents. Since 2005, all member countries except the so-called “least developed countries” have implemented TRIPS. The current global intellectual property regimen has been widely criticized for impeding access to medicines for LMIC populations. ^94^ Though there is a substantial literature on medical patents and alternative mechanisms to incentivize private investment in health research, this review did not aim to capture that literature. 

***3.2.4. Obligations of specific actors*.** Different actors play different roles in deciding what health research is conducted. These differences can affect what obligations they have or how the ethical considerations described above apply to their specific context. For example, it may be thought that national funding bodies have special obligations to their domestic populations, or that scientists working on a particular disease have special obligations to patients with that disease.

Researchers themselves have considerable discretion concerning the direction their research takes. It is true that they are constrained by their expertise, the parameters of grants, and various professional expectations (such as what is needed for promotion and what top journals are willing to publish). Nevertheless, they get to make choices about exactly which projects they pursue, with which patient populations, and with which collaborators. Collectively, researchers can also influence many other entities that set research priorities, such as universities, funding organizations, and governments. Several authors point out the opportunities that researchers have to influence what research gets done and articulate specific duties that result, such as to engage in research to promote global justice, ^95^ to advocate for better distributions of research funds, ^96^ and to allocate their leftover biospecimens fairly and in ways that maximize social value. ^97^

The institutions that host and conduct research also face allocation decisions. For example, it is fairly common for clinical trial sites to host multiple trials that recruit from similar patient populations. A cancer center might host several studies enrolling patients with colorectal cancer and sometimes the same patient could be eligible for more than one of these studies. In such cases, Luke Gelinas *et al.* argue, the host institutions should prioritize among the competing studies. ^98^ Institutions hosting research studies also found themselves facing allocation questions during the Covid-19 pandemic. ^99^ There were many potential interventions to test under conditions of urgency, but limited capacity at each site. Moreover, in many countries no national body was able to coordinate the various projects, resulting in duplicative effort, underpowered studies, and other inefficiencies. ^100^

Finally, there has been considerable discussion of ethical questions relating to health research funders and funding mechanisms. One overarching issue concerns the goals at which funders should aim. Is it simply up to the funder which topics they prioritize or which populations they seek to benefit? Does it matter whether the funder is a public or a private entity? Those who have engaged this question have generally agreed that the allocation of health research funding is amenable to ethical analysis on the basis of who is expected to benefit and that both public and private funders have obligations in this regard. ^101^ Pierson and Millum go into detail on the role-specific obligations of governmental, multilateral, non-profit, and for-profit funders. They argue that all have obligations to support socially valuable research, albeit within certain constraints—for example, within the constraints of mission statements for non-profits and within the constraint of making reasonable returns for for-profit organizations:

A funder intending to create an optimal distribution of research resources would maximize social value. The general duties of an individual research funding organization will also tend to favor maximizing the social value of research, but special duties may lead them to deviate from this baseline principle of allocation. ^102^

A further impediment to achieving a globally optimal distribution of research resources is the lack of coordination among funders. There is no agreed-upon global research and development agenda and consequently no division of labor among funders to prioritize and pursue that agenda. ^103^

The discussion of funding mechanisms primarily addresses grants and alternatives to the current process of grant review. One topic that has elicited some recent attention is whether allocating grants on the basis of peer review is efficient. The process of writing grant applications is extremely time-consuming and the success rate for applications relatively low. Moreover, it is dubious whether the system of review actually selects the best projects for funding. Critics of the current system have proposed alternatives that would reduce the workload without—they claim—reducing the quality of the resulting science, including allocating funding according to voting by other scientists or via lottery. ^104^

Distinct from the question of whether funding should be allocated through the mechanism of competitive grants is how such grants programs should be structured in order that the resulting allocation of funds is ethically justified. Bridget Pratt and Adnan Hyder identified a number of characteristics of projects that are likely to promote global health equity, including being conducted with worse-off populations, being led by LMIC institutions and researchers, and involving collaboration or consultation with LMIC policy makers and disadvantaged groups. ^105^ Grant programs can incentivize projects with these characteristics by, for example, requiring applications to include them or heavily weighting them in the process of review. ^106^ Some authors raised ethical concerns about how current grant schemes are set up and existing grant review processes. Emilie S. Koum Besson describes ways in which epistemic injustices affect the types of projects and the applicants that are funded by Global North funders. ^107^ Among other problems, she argues that funders privilege transferable over contextualized knowledge, and undervalue local expertise and local ways of knowing. Meanwhile, Pierson and Millum examine the criteria used by the largest public and non-profit health research funders to evaluate grant applications and compare them to ethical principles for research priority setting. ^108^ They conclude that while most funders use concrete criteria to ensure that they fund high quality science, few “explicitly instruct reviewers to consider the magnitude of the health problem a research project addresses nor which populations will benefit.” ^109^

***3.2.5. Research topics*.** A small number of the documents in this review focused on the ethics of allocating research resources in the context of specific topics—that is, particular research areas. I already described ethical issues relating to rare and neglected diseases. Other areas that were discussed included how priorities are set for pediatric research, nutrition research, and autism spectrum disease (ASD) research. ^110^ In discussing how priorities for ASD research are set in Canada, Krahn and Fenton criticize the way that the condition is frequently conceptualized as simply “a serious childhood disease or as a set of conditions that profoundly diminish the relevant patient population.” ^111^ Instead, they propose that:

… seeing autism as a disability instead of seeing it as a disease or impairment allows for the possibility of interpreting this condition as indicative, not of the affected individuals’ deficits but of a society being structured in ways that fail to adequately respond to—or, even just accommodate—the particular needs of this group. ^112^

Krahn and Fenton suggest that moving away from an exclusively medical model of ASD would also change what types of research were regarded as priorities—for example, investing more into research on services to support adults with ASD to live independently.

Two articles engaged with research on aging. ^113^ Anti-aging research is liable to provoke concerns about distributive justice, since the primary beneficiaries are likely to be older people. People who already live to an old age are likely to be better off than those who die prematurely on other measures, plus they have already had more of a very important good—life. Research to extend life beyond the normal span has prompted particular concern in this regard, while research to improve quality of life in the elderly is broadly supported. 

Finally, two articles considered how priorities should be set for bioethics research. Rachel Fabi and Daniel Goldberg argue that bioethics funding overemphasizes ethical issues that arise from novel technologies, to the detriment of bioethics research into topics more important for securing social justice. ^114^ Bridget Pratt and Adnan Hyder make the case for more bioethics scholarship on how funders should allocate scarce resources for health research. ^115^ They identify key decision points that matter a great deal for how resources are distributed but where there is little bioethics scholarship.

## 4. Discussion

This review revealed discussion of a wide range of topics in the literature relating to ethics and health research priority setting. However, despite the range of topics, the literature is quite thin in comparison, say, to the literature on the ethics of health *care* priority setting. A substantial proportion of the current discussion focuses on questions of the *process* by which research priorities should be set and how more marginalized voices can be better included in that process. Writers largely agree on two overarching substantive criteria—aiming for a greater magnitude of benefits and improving equity—while disagreeing over many more detailed questions of substance. Moreover, the debates over process and debates over substantive criteria seem like they are mostly being carried out in isolation from one another. This is despite the fact that how a priority setting process is carried out will naturally have implications for the outcomes of that process, which may then be judged according to substantive criteria.

A number of gaps in the topics covered were salient, which may suggest promising directions for future work. First, there are a number of methods for carrying out formal priority setting exercises. Nevertheless, it would likely be beneficial to have methods that are adapted to different decision-makers and contexts. For example, it is not clear how the existing formal methods should be used by individual funders, by institutions like universities, or by individual researchers or research groups. Yet all of these actors set priorities. Nor is there guidance on how to carry out priority setting when one has limited time or severe budgetary constraints. Methods like the CHNRI method or the ENHR strategy require a substantial investment of time and resources, which may not be practical for some actors or cost-effective for others whose research budgets are not that large.

Second, there are still open questions concerning how and when stakeholders should be involved in priority setting. For example, while the role of patients and community members has (rightly) received a lot of attention, there is less analysis of the appropriate role of representatives of funders, policy makers, and clinicians. Further, it seems plausible that stakeholder involvement should differ depending on the nature of the science—one might expect that basic science research would be quite different from comparative-effectiveness research in this respect, for instance.

Third, in-depth discussion of process criteria is mostly limited to questions of stakeholder inclusion. Other process criteria might also merit critical debate. Several documents make reference to A4R and assume that its principles apply to health research priority setting. There is not yet much critical consideration of how health research priority setting differs from health care priority setting, nor how the many criticisms of A4R should be addressed in this context.

Fourth, in comparison to the rich literature on health care priority setting, the debate over substantive criteria seems to be still in its infancy. This review prompted many questions that have barely been touched on so far. For example, how should we conceptualize equity? How should a specific conception of equity be applied to research priority setting? Do the different ways of stating the twin substantive goals of priority setting make a difference? How should we take the uncertainty and serendipity of scientific progress into account? How should global and more local priorities be related to one another? The field appears ripe for the application of work done elsewhere on global justice, decolonization, and the like.

This narrative review has several potential shortcomings. It is possible that the search strategy missed important documents relating to ethics and health research priority setting. This could be true of documents that are not indexed in PubMed, GoogleScholar, or PhilPapers. It could also occur if there is a relevant literature that uses different terminology and so was missed. However, checking the references of publications included in this review did not suggest obvious gaps.

Due to the goals of the broader WHO project, this was not a systematic review and did not involve quantitative data. Inevitably, with a review like this there is a subjective element involved in identifying themes in the literature and describing those themes. Moreover, much of what is important in the ethics literature lies in the details of arguments, analyses, and methodologies, which an overview cannot convey without reaching an excessive length. Choices had to be made about how far to aim for completeness versus readability.

Finally, it is worth emphasizing again that this review did not aim to capture the literature on intellectual property, innovation, and access to medicines. It is widely acknowledged that the global intellectual property regime introduced by the TRIPS agreement has enormous effects on what health research gets conducted. There is also a substantial literature that criticizes TRIPS and similar IP protections on ethical grounds. However, despite the close connections, IP regimes have not generally been treated as part of priority setting and so that literature was not synthesized as part of this review.

## 5. Conclusions

Neither the practice of health research priority setting nor the associated academic literature are as developed as their siblings in health *care* priority setting. None of the major institutions who allocate scarce resources for research do so on the basis of a data-driven and ethically justified priority setting process. Nonetheless, as this review reveals, there is a rich literature on the ethics of health research priority setting, which is relevant to the actions of the many policy makers, funders, research institutions, researchers, and others whose decisions affect what health research is carried out around the world.

## Ethics and consent

Ethical approval and consent were not required.

## Data Availability

No data are associated with this article.

## Footnotes

^1^ Commission on Health Research for Development. *Health research: essential link to equity in development*. Oxford University Press, USA, 1990: xvii.

^2^ Ramsay, Sarah. “No closure in sight for the 10/90 health-research gap.” *The Lancet* 358.9290 (2001): 1348.

^3^ De Winter, Jan. “How to make the research agenda in the health sciences less distorted.” *THEORIA. An International Journal for Theory, History and Foundations of Science* 27.1 (2012): 75–93; Resnik, David B. “The distribution of biomedical research resources and international justice.” Developing world bioethics 4.1 (2004): 42–57; Viergever, Roderik F. “The mismatch between the health research and development (R&D) that is needed and the R&D that is undertaken: an overview of the problem, the causes, and solutions.” Global Health Action 6.1 (2013): 22450.

^4^ Cf. Millum, J. “Background paper: The ethics of health research priority setting.” (2023). Available at: https://www.gfbr.global.

^5^ Gelinas, Luke, *et al.* “When clinical trials compete: prioritising study recruitment.” Journal of Medical Ethics 43.12 (2017): 803–809.

^6^ Meyer, M. N., Gelinas, L., Bierer, B. E., Hull, S. C., Joffe, S., Magnus, D., ... & Lynch, H. F. (2021). “An ethics framework for consolidating and prioritizing COVID-19 clinical trials.” Clinical Trials, 18(2), 226–233.

^7^ Lomas, J., Fulop, N., Gagnon, D., & Allen, P. “On being a good listener: setting priorities for applied health services research.” The Milbank Quarterly, 81.3 (2003): 363–388.

^8^ Goold, Susan Dorr, *et al.* “Members of minority and underserved communities set priorities for health research.” The Milbank Quarterly 96.4 (2018): 675–705.

^9^ Fleurence, Rachael L., and David J. Torgerson. “Setting priorities for research.” Health policy 69.1 (2004): 1–10.

^10^ Kapiriri L., Arnold E., Campbell S., Kapata- Chanda P., Ngosa W. and Humainza B. Approaches to Health Research Priority Setting: A Reference Manual Synthesizing the Literature and Demonstrating the Potential Use of the Manual. Mcmaster University, 2017; Montorzi G, de Haan S, IJsselmuiden C. Priority Setting for Research for Health: a management process for countries. Council on Health Research for Development (COHRED), 2010; Terry *et al.* A systematic approach for undertaking a research priority setting exercise. Guidance for WHO staff. Geneva: World Health Organization, 2020.

^11^ Manafò, Elizabeth, *et al.* “Patient and public engagement in priority setting: a systematic rapid review of the literature.” *PloS one* 13.3 (2018): e0193579.

^12^ Ad Hoc Committee on Health Research Relating to Future Intervention Options. *Investing in Health Research and Development*. World Health Organization, Geneva, 1996 (Document TDR/Gen/96.1): xxvi–xxvii.

^13^ Rudan, Igor, *et al.* “Setting health research priorities using the CHNRI method: VII. A review of the first 50 applications of the CHNRI method.” *Journal of global health* 7.1 (2017).

^14^ Igor Rudan, Shams El Arifeen, Robert E. Black A Systematic Methodology for Setting Priorities in Child Health Research Investments (2006): 5.

^15^ Ghaffar A, Collins T, Matlin SA, Olifson S. The 3D combined approach matrix: an improved tool for setting priorities in research for health. Geneva: Global Forum for Health Research (2009): 14.

^16^ Ghaffar 2009: 42.

^17^ Turoff, Murray, and Harold A. Linstone. *The Delphi method: techniques and applications*. Addison-Wesley Publishing Company, Advanced Book Program (2002).

^18^ de Meyrick, Julian. “The Delphi method and health research.” *Health education* 103:1 (2003): 7–16.

^19^ Okello D, Chongtrakul P, COHRED Working Group on Priority Setting. *A manual for research priority setting using the ENHR Strategy*. Geneva: Council on Health Research for Development (2000).

^20^ Okello and Chongtrakul 2000: 10.

^21^ Okello and Chongtrakul 2000: 11.

^22^ Okello and Chongtrakul 2000: 3.

^23^ James Lind Alliance. *The James Lind Alliance Guidebook, Version 10* (2021): 5.

^24^ James Lind Alliance 2021: 10.

^25^ James Lind Alliance 2021: 8.

^26^ Terry, R. F., *et al.* “An analysis of research priority setting at the World Health Organization–how mapping to a standard template allows for comparison between research priority setting approaches.” *Health research policy and systems* 16.1 (2018): 1–11, at 2.

^27^ Yoshida, Sachiyo. “Approaches, tools and methods used for setting priorities in health research in the 21st century.” *Journal of global health* 6.1 (2016).

^28^ Tomlinson, Mark, *et al.* “A review of selected research priority setting processes at national level in low and middle income countries: towards fair and legitimate priority setting.” *Health Research Policy and Systems* 9.1 (2011): 1–7.

^29^ Reveiz L, Elias V, Terry RF, Alger J, Becerra-Posada F. Comparison of national health research priority setting methods and characteristics in Latin America and the Caribbean, 2002–2012. *Rev Panam Salud Publica*. 2013;34(1):1–13.

^30^ Staley and Hanley 2008: 8.

^31^ Grill, Christiane. “Involving stakeholders in research priority setting: a scoping review.” *Research involvement and engagement* 7.1 (2021): 1–18.

^32^ See, e.g., Montorzi, G., S. De Haan, and C. IJsselmuiden. “Priority Setting for Research for Health: a management process for countries.” Geneva: Council on Health Research for Development (COHRED) (2010); Nasser *et al.* Prioritization of Research. In: WHO guidance on research methods for health emergency and disaster risk management. Geneva: World Health Organization; 2021.

^33^ Montorzi *et al.* 2010.

^34^ JLA 2021.

^35^ Pratt, Bridget. “Towards inclusive priority setting for global health research projects: recommendations for sharing power with communities.” *Health Policy and Planning* 34.5 (2019): 346–357, at 346.

^36^ Pratt, Bridget, Maria Merritt, and Adnan A. Hyder. “Towards deep inclusion for equity-oriented health research priority setting: a working model.” *Social Science & Medicine* 151 (2016): 215–224.

^37^ Cartier, Yuri, *et al.* “Priority setting in public health research funding organisations: an exploratory qualitative study among five high-profile funders.” Health research policy and systems 16.1 (2018): 1–10; Kapiriri, Lydia, Corinne Schuster-Wallace, and Pascalina Chanda-Kapata. “Evaluating health research priority setting in low-income countries: a case study of health research priority setting in Zambia.” Health Research Policy and Systems 16.1 (2018): 1–12; Kapiriri, Lydia. “Stakeholder involvement in health research priority setting in low income countries: the case of Zambia.” Research involvement and engagement 4.1 (2018): 1–9.

^38^ Pratt, Bridget, Prashanth N. Srinivas, and Tanya Seshadri. “How is inclusiveness in health systems research priority setting affected when community organizations lead the process?” Health Policy and Planning (2022).

^39^ Pratt, Bridget. “Sharing power in global health research: an ethical toolkit for designing priority setting processes that meaningfully include communities.” International journal for equity in health 20.1 (2021): 1–11.

^40^ Dowhaniuk, N., Ojok, S., & McKune, S. L. (2021). Setting a research agenda to improve community health: An inclusive mixed-methods approach in Northern Uganda. Plos one, 16(1), e0244249; Goold, S. D., Danis, M., Abelson, J., Gornick, M., Szymecko, L., Myers, C. D., ... & Salman, C. (2019). Evaluating community deliberations about health research priorities. Health Expectations, 22(4), 772–784; Goold, Susan Dorr, *et al.* “Members of minority and underserved communities set priorities for health research.” The Milbank Quarterly 96.4 (2018): 675–705.

^41^ Groot, Barbara, *et al.* “What Patients Prioritize for Research to Improve Their Lives and How Their Priorities Get Dismissed Again.” International Journal of Environmental Research and Public Health 19.4 (2022): 1927.

^42^ Patient Protection and Affordable Care Act of 2010, Pub. L. No. 111–148, 124 Stat. 119 (2010). Subtitle D—Patient-Centered Outcomes Research.

^43^ Cargill, S. S., Baker, L. L., & Goold, S. D. (2017). Show me the money! An analysis of underserved stakeholders’ funding priorities in Patient Centered Outcomes Research domains. Journal of Comparative Effectiveness Research, 6(5), 449–459; Goold, Susan Dorr, *et al.* “Priorities for patient‐centered outcomes research: the views of minority and underserved communities.” Health services research 52.2 (2017): 599–615; Goold, Susan Dorr, *et al.* “Members of minority and underserved communities set priorities for health research.” The Milbank Quarterly 96.4 (2018): 675–705; Goold, S. D., Danis, M., Abelson, J., Gornick, M., Szymecko, L., Myers, C. D., ... & Salman, C. (2019). Evaluating community deliberations about health research priorities. Health Expectations, 22(4), 772–784.

^44^ Manafò, E., Petermann, L., Vandall-Walker, V., & Mason-Lai, P. (2018). Patient and public engagement in priority setting: a systematic rapid review of the literature. PloS one, 13(3), e0193579.

^45^ Lomas, J., Fulop, N., Gagnon, D., & Allen, P. (2003). On being a good listener: setting priorities for applied health services research. The Milbank Quarterly, 81(3), 363–388.

^46^ Rudan, I. “Global health research priorities: mobilizing the developing world.” Public Health 126.3 (2012): 237–240.

^47^ E.g., Okello 2000: 13f; Working Group on Priority Setting, Council on Health Research for Development (COHRED). “Priority setting for health research: lessons from developing countries.” Health policy and planning 15.2 (2000): 130–136, at 134; Yoshida, Sachiyo, *et al.* “Setting health research priorities using the CHNRI method: III. Involving stakeholders.” *Journal of global health* 6.1 (2016).

^48^ Tomlinson, Mark, *et al.* “A review of selected research priority setting processes at national level in low and middle income countries: towards fair and legitimate priority setting.” Health Research Policy and Systems 9.1 (2011): 1–7; Yoshida *et al.* 2016.

^49^ E.g., Bhaumik, Soumyadeep, *et al.* “Ethics and equity in research priority setting: stakeholder engagement and the needs of disadvantaged groups.” Indian J Med Ethics 12.2 (2015): 110–113; Goold, Susan Dorr, *et al.* “Members of minority and underserved communities set priorities for health research.” The Milbank Quarterly 96.4 (2018): 675–705.

^50^ E.g., Kapiriri, Lydia. “Stakeholder involvement in health research priority setting in low income countries: the case of Zambia.” Research involvement and engagement 4.1 (2018): 1–9.

^51^ E.g., Pratt, Bridget. “Research for Health Justice: an ethical framework linking global health research to health equity.” BMJ Global Health 6.2 (2020): e002921.

^52^ Kapiriri, Lydia, and Pascalina Chanda-Kapata. “The quest for a framework for sustainable and institutionalised priority setting for health research in a low-resource setting: the case of Zambia.” Health research policy and systems 16.1 (2018): 1–12; Pratt, Bridget, and Bebe Loff. “A framework to link international clinical research to the promotion of justice in global health.” Bioethics 28.8 (2014): 387–396.

^53^ E.g., Resnik, David B. “Setting biomedical research priorities: justice, science, and public participation.” Kennedy Institute of Ethics Journal 11.2 (2001): 181–204.

^54^ Groot, Barbara, *et al.* “What Patients Prioritize for Research to Improve Their Lives and How Their Priorities Get Dismissed Again.” International Journal of Environmental Research and Public Health 19.4 (2022): 1927.

^55^ Besson, Emilie S. Koum. “How to identify epistemic injustice in global health research funding practices: a decolonial guide.” *BMJ global health* 7.4 (2022): e008950.

^56^ Ward, Claire Leonie, *et al.* “Defining Health Research for Development: The perspective of stakeholders from an international health research partnership in Ghana and Tanzania.” Developing world bioethics 18.4 (2018): 331–340.

^57^ Parker, Michael, and Patricia Kingori. “Good and bad research collaborations: researchers’ views on science and ethics in global health research.” *PloS one* 11.10 (2016): e0163579.

^58^ Kok, Maarten Olivier, *et al.* “Towards fair and effective North–South collaboration: realising a programme for demand-driven and locally led research.” Health Research Policy and Systems 15.1 (2017): 1–17.

^59^ E.g., Tomlinson, Mark, *et al.* “A review of selected research priority setting processes at national level in low and middle income countries: towards fair and legitimate priority setting.” Health Research Policy and Systems 9.1 (2011): 1–7; Tong, Allison, *et al.* “Reporting guideline for priority setting of health research (REPRISE).” BMC medical research methodology 19.1 (2019): 1–11.

^60^ E.g., Montorzi *et al.* 2010; Pratt *et al.* 2016; Rudan, Igor, *et al.* “Setting priorities in global child health research investments: assessment of principles and practice.” Croatian medical journal 48.5 (2007): 595.

^61^ Daniels, Norman. “Accountability for reasonableness: Establishing a fair process for priority setting is easier than agreeing on principles.” *Bmj* 321.7272 (2000): 1300–1301.

^62^ E.g., Callahan, Daniel. “Shaping biomedical research priorities: the case of the National Institutes of Health.” *Health Care Analysis* 7.2 (1999): 115–129; May, Thomas. “Funding agendas: has bioterror defense been over-prioritized?” The American Journal of Bioethics 5.4 (2005): 34–44.

^63^ E.g., Fleurence, Rachael L., and David J. Torgerson. “Setting priorities for research.” Health policy 69.1 (2004): 1–10; Rudan, I. “Global health research priorities: mobilizing the developing world.” Public Health 126.3 (2012): 237–240; Viergever, Roderik F., *et al.* “A checklist for health research priority setting: nine common themes of good practice.” Health research policy and systems 8.1 (2010): 1–9.

^64^ Barsdorf, N., & Millum, J. (2017). “The social value of health research and the worst off.” Bioethics, 31(2), 105–115, at 107.

^65^ Nuyens, Yvo. “Setting priorities for health research: lessons from low-and middle-income countries.” Bulletin of the World Health Organization 85 (2007): 319–321, at 321.

^66^ Persad, Govind, and Jessica du Toit. “The case for valuing non-health and indirect benefits.” In: Norheim, Ole F., Ezekiel J. Emanuel, and Joseph Millum, eds. *Global health priority-setting: beyond cost-effectiveness*. Oxford University Press (2019).

^67^ E.g., Mohindra *et al.* 2012: 840; Nasser, M., Ueffing, E., Welch, V., & Tugwell, P. (2013). An equity lens can ensure an equity-oriented approach to agenda setting and priority setting of Cochrane Reviews. Journal of clinical epidemiology, 66(5), 511–521, at 511; Pratt *et al.* 2016.

^68^ E.g., Bailey, T.C., & Sugarman, J. (2013). Social justice and HIV vaccine research in the age of pre-exposure prophylaxis and treatment as prevention. Current HIV research, 11(6), 473–480; Barsdorf and Millum 2017.

^69^ E.g., Pratt, Bridget. “Research for Health Justice: an ethical framework linking global health research to health equity.” BMJ Global Health 6.2 (2020): e002921.

^70^ Wenner, D.M. (2018) “The Social Value Requirement in Research: From the Transactional to the Basic Structure Model of Stakeholder Obligations,” Hastings Center Report 48(6): 25–32, at 31. See, also, Wenner, D.M. (2021) “The Social Value of Research and Health Research Priority setting”. In Ana S. Iltis and Douglas MacKay (eds.) *The Oxford Handbook of Research Ethics*.

^71^ Wenner 2018: 32.

^72^ E.g., Terry *et al.* 2020: 16; Viergever *et al.* 2010: 9.

^73^ Fleurence and David Torgerson 2004.

^74^ Claxton, Karl, and John Posnett. “An economic approach to clinical trial design and research priority‐setting.” Health economics 5.6 (1996): 513–524.

^75^ Gericke, C. A., Annette Riesberg, and Reinhard Busse. “Ethical issues in funding orphan drug research and development.” Journal of Medical Ethics 31.3 (2005): 164–168; Viergever, Roderik F. “The mismatch between the health research and development (R&D) that is needed and the R&D that is undertaken: an overview of the problem, the causes, and solutions.” Global Health Action 6.1 (2013): 22450.

^76^ For data on neglected disease research, see Policy Cures Research. *G-FINDER*. Available at: https://www.policycuresresearch.org/g-finder/.

^77^ Gericke *et al.* 2005.

^78^ Institute of Medicine 2009. *Initial National Priorities for Comparative Effectiveness Research*. Washington, DC: The National Academies Press. Chapter 4. “The Criteria and Process for Setting Priorities”, at 79.

^79^ Gammie, Todd, Christine Y. Lu, and Zaheer Ud-Din Babar. "Access to orphan drugs: a comprehensive review of legislations, regulations and policies in 35 countries." *PloS one*10.10 (2015): e0140002.

^80^ For a long list of these critics, see Millum, Joseph. “Should health research funding be proportional to the burden of disease?” *Politics, Philosophy & Economics* (2022): 1470594X221138729.

^81^ This view is adopted with respect to the global allocation of health research resources in Flory, James H., and Philip Kitcher. “Global health and the scientific research agenda.” Philosophy & Public Affairs 32.1 (2004): 36–65.

^82^ Millum 2022: 19.

^83^ Pang, Tikki. “Global health research: changing the agenda.” In *Global Health and Global Health Ethics*, ed. Solomon Benatar and Gillian Brock. Cambridge University Press (2011): 285–292.

^84^ Krahn, Timothy M., and Andrew Fenton. “Funding priorities: Autism and the need for a more balanced research agenda in Canada.” Public Health Ethics 5.3 (2012): 296–310.

^85^ E.g., Okello and Chongtrakul 2000; Rudan *et al.* 2006.

^86^ Bloom, B. R., Michaud, C. M., La Montagne, J. R., & Simonsen, L. (2006). Priorities for global research and development of interventions. Disease Control Priorities in Developing Countries. 2nd edition, at 112.

^87^ De Winter 2012; Resnik 2004; Viergever 2013.

^88^ Mohindra, K. S., *et al.* “Towards the next generation of public health research in India: a call for a health equity lens.” *J Epidemiol Community Health* 66.9 (2012): 839–842; Pratt, Bridget, and Adnan A. Hyder. “Fair resource allocation to Health Research: priority topics for bioethics scholarship.” Bioethics 31.6 (2017): 454–466.

^89^ Besson 2022; Parker and Kingori 2016; Ward 2018.

^90^ Nuyens, Yvo. “Setting priorities for health research: lessons from low-and middle-income countries.” Bulletin of the World Health Organization 85 (2007): 319–321.

^91^ Pratt, Bridget, Mark Sheehan, Nicola Barsdorf, and Adnan A. Hyder. “Exploring the ethics of global health research priority setting.” BMC medical ethics 19, no. 1 (2018): 1–11.

^92^ Pang 2011; Pratt and Loff 2014; Pratt, Bridget, and Adnan A. Hyder. “Designing research funding schemes to promote global health equity: an exploration of current practice in health systems research.” Developing World Bioethics 18.2 (2018): 76–90.

^93^ Sterckx, Sigrid. “Patents and access to drugs in developing countries: an ethical analysis.” *Developing World Bioethics* 4.1 (2004): 58–75.

^94^ E.g., Oprea, Liviu, Annette Braunack-Mayer, and Christian A. Gericke. “Ethical issues in funding research and development of drugs for neglected tropical diseases.” Journal of medical ethics 35.5 (2009): 310–314.

^95^ Pratt, Bridget. “Research for Health Justice: an ethical framework linking global health research to health equity.” BMJ Global Health 6.2 (2020): e002921.

^96^ Flory and Kitcher 2004: 61.

^97^ Pierson, Leah, *et al.* “Allocation of scarce biospecimens for use in research.” Journal of Medical Ethics 47.11 (2021): 740–743.

^98^ Gelinas, Luke, *et al.* “When clinical trials compete: prioritising study recruitment.” Journal of Medical Ethics 43.12 (2017): 803–809.

^99^ Meyer, M. N., Gelinas, L., Bierer, B. E., Hull, S. C., Joffe, S., Magnus, D., ... & Lynch, H. F. (2021). An ethics framework for consolidating and prioritizing COVID-19 clinical trials. Clinical Trials, 18(2), 226–233.

^100^ Glasziou, Paul P., Sharon Sanders, and Tammy Hoffmann. “Waste in covid-19 research.” *Bmj* 369 (2020).

^101^ Flory and Kitcher 2004; Pierson and Millum 2018; Wenner 2018.

^102^ Pierson and Millum 2018: 15.

^103^ Viergever, Roderik F. “The mismatch between the health research and development (R&D) that is needed and the R&D that is undertaken: an overview of the problem, the causes, and solutions.” Global Health Action 6.1 (2013): 22450.

^104^ Barnett, Adrian G., *et al.* “Using democracy to award research funding: an observational study.” Research Integrity and Peer Review 2 (2017): 1–9; Fang, Ferric C., and Arturo Casadevall. “Research funding: The case for a modified lottery.” MBio 7.2 (2016): e00422–16; Roumbanis, Lambros. “Peer review or lottery? A critical analysis of two different forms of decision-making mechanisms for allocation of research grants.” Science, Technology, & Human Values 44.6 (2019): 994–1019.

^105^ Pratt, Bridget, and Adnan A. Hyder. “Designing research funding schemes to promote global health equity: an exploration of current practice in health systems research.” Developing World Bioethics 18.2 (2018): 76–90.

^106^ Pratt, Bridget. “Research for Health Justice: an ethical framework linking global health research to health equity.” BMJ Global Health 6.2 (2020): e002921.

^107^ Besson 2022.

^108^ Leah Pierson & Joseph Millum (2022) Health Research Priority Setting: Do Grant Review Processes Reflect Ethical Principles?, Global Public Health, 17:7, 1186–1199

^109^ Pierson and Millum 2022: 1196.

^110^ Hawwash, D., Pinxten, W., Bonn, N. A., Verstraeten, R., Kolsteren, P., & Lachat, C. (2018). Perspective: consideration of values when setting priorities in nutrition research: guidance for transparency. Advances in Nutrition, 9(6), 671–687; Krahn and Fenton 2012; Pellicano, Liz. “A future made together: new directions in the ethics of autism research.” Journal of research in special educational needs 14.3 (2014): 200–204; Rudan, Igor, *et al.* “Setting priorities in global child health research investments: assessment of principles and practice.” Croatian medical journal 48.5 (2007): 595.

^111^ Krahn and Fenton 2012: 304.

^112^ Krahn and Fenton 2012: 304.

^113^ Farrelly, Colin. “Aging research: priorities and aggregation.” Public Health Ethics 1.3 (2008): 258–267; Lesser, Harry. “Priorities in the Use of Research into Ageing.” Health Care Analysis 13 (2005): 53–58.

^114^ Fabi, Rachel, and Daniel S. Goldberg. “Bioethics, (funding) priorities, and the perpetuation of injustice.” The American Journal of Bioethics 22.1 (2022): 6–13.

^115^ Pratt, Bridget, and Adnan A. Hyder. “Fair resource allocation to Health Research: priority topics for bioethics scholarship.” Bioethics 31.6 (2017): 454–466.

