# Supplementary material for: Ethics and health research priority setting: a narrative review
Source: Wellcome Open Res. 2024 Apr 17;9:203. [Version 1] doi: 10.12688/wellcomeopenres.21182.1 (PMC11234074; doi:10.12688/wellcomeopenres.21182.1)
Supplement: Peer review history

# Reviewer report: Reviewer response for version 1

**DOI:** 10.21956/wellcomeopenres.23427.r87132

**Published:** 2024-07-09

**Authors:** Lars Sandman

**Affiliations:** 1 Linköping University, National Centre for Priorities in Health, Linköping, Sweden

Thank you for the opportunity to review this narrative review about ethics and health research priority setting. I find the article addressing an important topic in a well-researched and well-written way. It provides a very good overview and starting point for a further discussion on the, to my mind, somewhat neglected topic of health research priorities. I only have a few minor, but I still think, somewhat essential aspects to consider.

 On page 4 the author lists three questions to be answered when considering priority setting of health research. One aspect I miss there, which is common when considering health priorities, is assessment of the outcomes of the priority exercise. Of course, to some extent depend on the criteria set up for priority setting, we want to know to what extent the fulfilment of these criteria was fulfilled through our exercise.

 In the Discussion, when considering the involvement of stakeholders, I miss a discussion about the role of different patient representatives. This is a challenge in health priorities, since different priority decisions will benefit and disadvantage different patient groups. Normally, at least when considering one health technology at the time, all these patients that might suffer the opportunity cost if the technology is granted reimbursement (or something of sorts), might not be possible to bring to the table. In this case, when actually prioritizing between different research proposal, it is at least principally possible to bring all different stakeholder to the table. However, this raises a number of interesting and difficult issue of their role, who should be chosen if not all can be brought to the table etc.

 In the Discussion, I also lack a discussion on how the transparent priority setting of health research should relate to the important value of academic freedom and allowing researchers to freely (well, within a number of constraints but still…) choose their research questions and methods.

Is the review written in accessible language?

Yes

Are all factual statements correct and adequately supported by citations?

Yes

Are the conclusions drawn appropriate in the context of the current research literature?

Yes

Is the topic of the review discussed comprehensively in the context of the current literature?

Yes

Reviewer Expertise:

Health care ethics, priority setting ethics

I confirm that I have read this submission and believe that I have an appropriate level of expertise to confirm that it is of an acceptable scientific standard.

# Reviewer report: Reviewer response for version 1

**DOI:** 10.21956/wellcomeopenres.23427.r82598

**Published:** 2024-06-20

**Authors:** Wim Pinxten

**Affiliations:** 1 Hasselt University, Hasselt, Belgium

The article concerns a narrative review of health research priority setting. The topic is timely and relevant. The paper is well structured and explains the complex problems of priority setting, the equally complex context in which it takes place, and six different approaches in a way that is easy to understand for a broad readership with various disciplinary backgrounds.

 A few minor comments: 

 -In the introduction, the paper states that which research gets done does predictably affect who will benefit from new research". This does not always need to be the case, and later in the paper, it is recognized  that sometimes much depends on scientific serendipity and cannot just be predicted in advance. 

 -In the paragraph on rare diseases and orphan drugs, I would suggest to change "orphan diseases" into "rare diseases", as the designation "orphan" generally is attributed to the drugs, not to the rare disease. In the discussion related to rare diseases, it would be relevant to mention that these diseases are not only rare, but typically also have a high and unmet disease burden. Besides, the ethical issues related to neglected diseases are quite different than those related to rare diseases. 

 More generally, there are several other issues that could be added to the discussion, including the risk of abandonment (when lacking all traits that could move you up on the priority ladder), the freedom to advocate for ones priorities, conflicts of interests, etc... However, the uptake of these issues in the discussion is not essential.

Is the review written in accessible language?

Yes

Are all factual statements correct and adequately supported by citations?

Yes

Are the conclusions drawn appropriate in the context of the current research literature?

Yes

Is the topic of the review discussed comprehensively in the context of the current literature?

Yes

Reviewer Expertise:

Medical Ethics

I confirm that I have read this submission and believe that I have an appropriate level of expertise to confirm that it is of an acceptable scientific standard.
